# Critical assessment of methods of protein structure prediction—Round VII

**DOI:** 10.1002/prot.21767

**Published:** 2007-10-05

**Authors:** John Moult, Krzysztof Fidelis, Andriy Kryshtafovych, Burkhard Rost, Tim Hubbard, Anna Tramontano

**Affiliations:** 1 Center for Advanced Research in Biotechnology, University of Maryland Biotechnology InstituteRockville, Maryland 20850; 2 Genome Center, University of California, DavisCalifornia 95616; 3 Department of Biochemistry and Mol Biophysics, CUBIC, C2B2, NESG, Columbia UniversityNew York, New York 10032; 4 Wellcome Trust Sanger Institute, Wellcome Trust Genome CampusCambridgeshire CB10 1SA, United Kingdom; 5 Istituto Pasteur – Fondazione Cenci Bolognetti, University of Rome “La Sapienza”P.le Aldo Moro, 5, 00185 Rome, Italy

**Keywords:** protein structure prediction, community wide experiment, CASP

## Abstract

This paper is an introduction to the supplemental issue of the journal PROTEINS, dedicated to the seventh CASP experiment to assess the state of the art in protein structure prediction. The paper describes the conduct of the experiment, the categories of prediction included, and outlines the evaluation and assessment procedures. Highlights are improvements in model accuracy relative to that obtainable from knowledge of a single best template structure; convergence of the accuracy of models produced by automatic servers toward that produced by human modeling teams; the emergence of methods for predicting the quality of models; and rapidly increasing practical applications of the methods.

## INTRODUCTION

This issue of PROTEINS is devoted to papers reporting the outcome of the seventh community wide experiment to assess methods of protein structure prediction (CASP7), and related activities. There have been six previous CASP experiments, at 2 year intervals from 1994 through 2004, and these were reported in previous supplemental issues of PROTEINS.^1^–^6^ A separate description of the CASP7 experiment is also available.^7^

The primary goals of CASP are to establish the capabilities and limitations of current methods of modeling protein structure from sequence, to determine where progress is being made, and to determine where the field is held back by specific bottlenecks. With a substantial history of CASP experiments in place, bottlenecks and progress have become more important. Methods are assessed on the basis of the analysis of a large number of blind predictions of protein structure.

This paper outlines the structure and conduct of the experiment, and is followed by descriptions of the numerical analysis methods^8^ and of the CASP7 target proteins.^9^ There are papers by the assessment teams in each of the three-dimensional prediction categories template-free modeling,^10^ template-based modeling,^11^ and high-accuracy structure modeling,^12^ followed by five papers from some of the more successful modeling teams submitting in these categories. These are followed by an assessment of the current performance of automated structure prediction servers.^13^ The papers also describe assessment in the five structure related modeling areas covered in CASP7. For the third time, prediction of disordered regions was included,^14^ an area that continues to grow in experimental importance.^15^ Prediction of the boundaries of structural domains is also included,^16^ as it was in CASP6.^17^ Correctly identifying domain boundaries is often crucial to the modeling of large structures, and is also often key to successful experimental expression of multidomain proteins. The third paper covers the prediction of three-dimensional contacts between residues.^18^ A portion of the CASP prediction community is convinced that in the long run, this technique will make a major contribution to three-dimensional modeling methods, so it continues to be included, although there is little sign of progress. The fourth paper deals with the prediction of the function of proteins.^18^ Function prediction was also included in CASP6, and although initial evaluation was complicated by lack of experimental data,^19^ eventually a clearer and useful picture emerged.^20^ The final paper in this set of five describes assessment of model quality prediction.^21^ Quality prediction has always been included in CASP, but has not received much attention until now. If the structure modeling field is to be taken seriously, it is critical that we develop methods for reliably informing users how accurate our models are or are not. There is also a paper describing the results from one of the most effective quality prediction methods.^22^ The last paper in the issue is once again a survey of progress in the three-dimensional modeling categories since the last CASP, in the context of performance over all CASPs.^23^ As always, the assessors' papers are probably the most important in the whole issue, and describe the state of the art as they found it in CASP7.

## THE CASP7 EXPERIMENT

The structure of the experiment was very similar to that of the earlier ones, with a prediction season of about 3 months, and three main steps:

1. Information about “soon to be solved” structures was collected from the experimental community and passed on to the prediction community. As discussed later, in CASP7, nearly all targets were obtained from the Structural Genomics community. Target information was made available through the CASP web site, and sent directly to registered servers.
2. Prediction teams deposited models of the structures before the experimental results were public. For human prediction teams, deposition was required by a specified deadline. Deadlines were considerably tighter in CASP7 than previously, usually with a 3 week prediction window, to reduce loss of targets through leakage of experimental information. Servers were required to respond within 48 h.
3. The models were compared with experiment, using numerical evaluation techniques and human assessment, and a meeting was held to discuss the significance of the results.

## MANAGEMENT AND ORGANIZATION

CASP is a complicated process, requiring very careful data management and security, and mechanisms to ensure that the prediction community is informed and consulted. The principal components are:

- Organizers. The authors of this paper, responsible for all aspects of the organization of the experiment and meeting.
- The FORCASP web site (http://www.FORCASP.org). FORCASP provides a forum where members of the prediction community may discuss aspects of the CASP experiment.
- Predictors' meeting at Asilomar. During each CASP conference, there is a predictors' meeting with votes on issues of CASP policy, particularly major changes and extensions of the CASP process.
- Independent assessors. The independent assessors have primary responsibility for judging the quality of the predictions received, and commenting on the current state of the art. Assessors are provided with numerical analysis data generated using approved procedures, and may also add their own numerical methods.
- Protein Structure Prediction Center. The prediction center is responsible for all data management aspects of the experiment, including the distribution of target information, collection of predictions, generation of numerical evaluation data, developing tools for data analysis, data security, and maintenance of a web site where all data are available. Details of these aspects of the experiment are described in Kryshtafovych *et al*.^8^ In 2005, the center moved from Lawrence Livermore Lab to UC Davis.

## COLLECTION OF TARGETS

The CASP process relies on obtaining a supply of targets to be used as prediction goals by participating groups. These targets must be of proteins where the experimental structure is not yet public, but for which the structure will be available shortly. In early CASPs, targets were identified by large scale canvassing of individual X-ray crystallography and NMR spectroscopy groups around the world. That process was very labor intensive, since typically a group was only able to provide a single target, and some targets were lost because they were not solved in time. By CASP6, structural genomics projects provided more than half of the targets for the experiment. In CASP7, the vast majority of targets were from this source and for the largest contributors (The NIH PSI large scale centers http://www.nigms.nih.gov/Initiatives/PSI and the Structural Genomics Consortium http://www.sgc.utoronto.ca/), the target collection procedure was formalized, with a 3-week hold in the PDB before release of the experimental structures. This procedure had several advantages: (i) all target structures were solved beforehand, so there were no losses of that type; (ii) there were very few leaks of structural information (a particular problem in CASP6); (iii) there was a smooth flow of information among the SG centers, the PDB, and the Prediction Center; and, (iv) because of the high throughput in structural genomics, we were able for the first time to reach our long time goal of 100 prediction targets.

One hundred and four protein sequences were released for prediction. Details of 102 structures were obtained from the experimental community. Information on four of these targets was released prematurely, causing them to be cancelled. Additionally, three targets were canceled by the assessors because of poor structure quality, leaving 95. These were divided into domains, each of which was treated as a separate target for assessment purposes in the three-dimensional structure and contact prediction categories. In all, 123 domains were included.

## CATEGORIES OF PREDICTION

The quality of a structure model depends on how much information from already known structures can be used—at one extreme, models competitive with experiment can be produced for proteins with sequences very similar to that of a known structure. At the other, models for proteins with no detectable sequence or structure relationship to one of known structure are only rarely of high quality. In all previous CASPs, targets were divided into three broad categories, reflecting the likely quality of the models. These categories were (i) comparative modeling, where a related structure or structures for use as a template could be identified using a simple BLAST search; (ii) fold recognition, where more sophisticated methods could identify templates; and (iii) free modeling, for targets where no relationship to a known structure could be found. In CASP7, the first two categories were merged to include all template-based modeling, with one assessment team looking at the full range. A new category, high-accuracy modeling, was introduced, containing those template-based models where problems of alignment and template coverage were expected to be sufficiently small that the accuracy of resulting models should be competitive with experimental structures. The assessment team for this area looked at more detailed features, particularly side chain accuracy, accuracy of modeling of nonstructurally conserved regions, accuracy of regions most relevant to function, and usefulness of the models for molecular replacement. The free modeling, or template-free category, remained unaltered, containing targets which the assessors judged as having no domain level templates available (“new folds”) or for which it was clear that template-free methods produced the best results. Important evaluation criteria in the template-free category are the fraction of the structure which is predicted below a specified error level, and recognition of success in identifying general architecture.

## LEVEL OF PARTICIPATION

As always, a high level of participation from the prediction community is critical to the success of the experiment. Overall participation has increased from 35 groups in CASP1, then 70, 98, 163, 215, 228, and in CASP7, 253.

## COLLECTING AND VALIDATING PREDICTIONS

There were a total of 63,717 models deposited in CASP6, of which 48,339 are three-dimensional coordinate sets. A further 3,816 are alignments which were converted into coordinates for assessment. The remainder are residue–residue contacts (1,561), domain assignments (2,515), disorder predictions (1,801), function predictions (1,930), and three-dimensional model quality predictions (3,228). As usual, all predictions were required to be submitted to the Prediction Center in a machine readable format. Accepted submissions were issued an accession number that served as the record that a prediction had been made by a particular group on a particular target. Human predictions were submitted through the web interface, or by email. A final acceptance time was established for predictions on each target, determined by the expected release date of the experimental structure, or other factors. In CASP7, this was usually 3 weeks, with extension to 6 weeks in some cases. Target queries were sent to servers directly from the CASP distribution server and the returned models were immediately processed by the CASP verification software. Servers had 48 h in which to respond. The prediction season ran from May 10th until August 7th. As previously, each prediction group was limited to a maximum of five models per target, and were instructed that most emphasis would be placed on the model they designated as the best (referred to as “model 1”).

## NUMERICAL EVALUATION OF PREDICTIONS

In CASP, the accuracy of three-dimensional structure models are primarily evaluated using two metrics. One is GDT_TS, a multithreshold measure related to the difference in position of main chain Cα atoms between a model and the corresponding experimental structure.^24^ The other is alignment accuracy, AL0, showing how well the assigned amino acid positions accord with those in the experimental structure. Both these measures have been stable for several CASPs, though experiments with alternatives continue. In CASP7, a finer grain measure of main chain accuracy, GDT_HA, was introduced (thresholds of 0.5, 1, 2 and 4 Å, as opposed to 1, 2, 4, and 8 in GDT_TS), with the intent of better capturing any small but significant improvements in high-accuracy modeling. Both GDT measures were used by the assessors for the analysis of template-based modeling. As in previous CASPs, the assessors for the template-free category found that GDT_TS is useful for shortlisting the most noteworthy models, but that visual inspection is necessary to obtain a final ranking.^10^ An alternative measure of alignment accuracy, based on a dynamic programming procedure (SWALI),^23^ was used in part of the analysis to establish maximum possible alignability between the target and a single template. The Prediction Center also provided results from DALI, MAMMOTH, and ACE software to the assessors to facilitate their structural analysis. The assessors also employed their own measures and approaches to complement the conventional CASP ones. In disorder and domain evaluation, evaluation measures were the same as CASP6, with some refinements. The measure used for contact evaluation was altered, and that does affect the apparent usefulness of the methods.^18^ New criteria have been introduced in function^25^ and quality prediction^21^ assessment.

## ASSESSMENT

The numerical evaluation metrics, though critical, are not generally sufficient to draw final conclusions about the quality and usefulness of modeling methods. A key principle of CASP is that primary responsibility for assessing the significance of the results is placed in the hands of independent assessors. This continues to be a major source of insight and innovation in CASP, as well as ensuring that organizer biases are not imposed on the outcome. In CASP7, we saw multiple examples of the value of this procedure. Randy Read, the high-accuracy category assessor, introduced performance in molecular replacement as a very practical test of model usefulness and quality. Torsten Schwede, the template-based modeling category assessor, introduced a new hydrogen bond conservation score for his analysis and provided a new view of model quality relative to information in a single best template, revealing that many models are of higher quality by this measure than previously appreciated. Neil Clarke, the template-free category assessor, performed a rigorous evaluation of GDT_TS versus visual ranking of model quality, showing where the differences arise, and how many highly ranked GDT_TS models must be considered. He also introduced a new, contact map overlap score and changed the criteria for evaluating contact predictions, putting the usefulness of these methods in a new light. Excellent analysis was also performed by the assessors of function (Alfonso Valencia), domains (Michael Tress), and disorder (Lorenza Bordoli). As in other recent CASPs, all the assessors have taken care not to push the interpretation of the results beyond the point justified by statistical considerations.

## MEETINGS, WEB SITE, AND PUBLICATIONS

For the first time, there was a one day “Between CASPs” public meeting, held in New York in May, 2006. The aim of this and future such meetings was to bring the CASP results to a less specialized audience than would otherwise attend the regular workshops. The first CASP7 planning meeting, attended by the assessment teams for CASP7 and the previous assessors, was held in association with the New York event. Following the closing of the prediction season, a second planning meeting was held, at which the assessors presented their results to each other and to the organizers. As always, prediction team identities were hidden from the assessors until after those presentations, to avoid ranking bias.

The meeting to discuss the outcome of the experiment was held at the Asilomar Conference Center, site of all but one of the CASP meetings so far. The format of the meeting was again changed from previous CASPs. The first day was devoted to the five non-three-dimensional modeling areas, reflecting their increasing importance in CASP. On the second day, we heard presentations from the three assessors in the three-dimensional modeling categories, ending with a discussion of those results. The motivation for grouping these in a single session was that the methods and results in the different categories have become increasingly overlapping. In particular, several of the best groups were near top performers in two or more categories. On the third day, there were talks from a number of the more successful prediction teams, selected by the assessors. We did not repeat the CASP6 procedure of devoting a day to promising methods. That was generally not considered a success—as one predictor put it “I don't come to CASP to listen to talks about things that don't work”. Nevertheless, increasing the emphasis on developing new methods remains a major goal of the CASP organizers, and attention is now focused on the “Off-CASP” experiments, and “CASP challenges,” discussed later. The final half day of the meeting had talks on actual and potential applications areas for modeling—structure modeling in cancer, providing a modeling resource to the biology community, protein design, cryoelecton microscopy, structure from cross-linking, and low angle X-ray scattering. There was also an afternoon session with presentations by physicists working in the area of protein folding. Another goal of the organizers is to promote more interaction with this community, because it is clear that to advance further, more physics must be brought back into modeling. However, although there were some excellent presentations, it was clear from the discussion that there is a still major cultural difference between these communities, especially with regard to the value of rigorous, large scale testing of methods. There were a number of other sessions and group meetings. The full program can be found on the Prediction Center web site.

This issue of PROTEINS is the official report of the CASP7 experiment. Predictors submitting papers were urged to concentrate on what went right, what went wrong, and where possible, to explain why, and what they learned as a result. Because of space limitations, details of the methods are often absent, and readers are requested to turn to the references for more information. All of the prediction and assessment papers in this issue have been peer-reviewed. The CASP web site (http://predictioncenter.org) provides extensive details of the targets, the predictions, and the numerical analyses. Discussions of a number of issues can also be found on the FORCASP site (http://www.FORCASP.org). There are many possible views that may be taken of the results and the interested reader is encouraged to consult other sources, for alternative points of view.

## PROGRESS IN CASP7

CASP has now been in operation for 12 years and, as previously discussed,^26^ there has been an enormous amount of progress over that time. The quality of typical mid-range template models has approximately doubled since CASP1^23^ as measured by the CASP GDT_TS standard and template-free modeling has evolved from near-random to producing quite impressive models for some smaller proteins. Although cumulative progress is very impressive, changes between any successive pair of CASP experiments has often been modest overall, but usually with a few notable advances. That was again the case between CASP6 and CASP7. Four advances in particular stand out:

1. In template-based modeling, a majority of the best models for each target are more accurate than a model that could be produced from knowledge of the single closest experimental structure. The absolute value of the improvement over template is often rather small, but this is still a considerable achievement. There are also several impressive cases where the “added value” is about 10% in GDT_TS over that of the best template. Further, the fraction of models for which this is the case has been increasing steadily over the last three experiments—seven cases of over 10% improvement in CASP7, four cases in CASP6 and none in CASP5. This “added value” over incorporating all information in a best template (itself very nontrivial) has been a long time goal of CASP.
2. There is evidence that added value over a single template model is being achieved by three different methods. There are examples in the CASP7 results of combining information from two or more templates; from using template-free procedures to model parts of a structure not available from any template; and of the use of sophisticated all-atom refinement procedures to move structures away from a template-based model and towards the experimental result. Although these methods are far from universally effective, it is encouraging to see clear evidence of their potential. This is particularly true of refinement, which has been a focus in CASP now for several experiments.
3. The accuracy of models produced by automatic servers is moving close to that of humans, and the gap has closed substantially over the last three CASPs. This is the case even though human groups are provided with the set of server results in CASP, and usually use these as a starting model. Server/human convergence is particularly significant since the amount of effort required for a human group to produce a model is too great for application to the enormous number of available sequences. Further, the availability of many of the servers puts high-quality modeling tools into the hands of the general biologist.
4. Methods for estimating model accuracy, while still in need of much development, have been shown to be already useful.^21^ At the moment, this is most true for model quality rankings produced by some metaservers, where the results are based on commonalities between models from multiple, previously-calibrated sources.

In contrast to these developments, standard measurements of overall progress (GDT_TS and alignment accuracy) show only modest change between CASP6 and CASP7. Also, the new template-free modeling methods, which caused such excitement starting in CASP4, seem to have run out of steam for the moment. Once again, there were several very impressive models for small targets, but no detectable overall advance. It should be noted that there are relatively few of these targets, so small improvements are hard to spot.

## THE EVOLUTION OF CASP

The increased emphasis on aspects of structure modeling beyond simple structure accuracy in CASP reflects a more general evolution of the field away from a rather irrelevant academic pursuit to a very practical and applied area. In three-dimensional modeling, the greatly increased set of experimental structures allows a higher fraction of structures to be modeled based on a close template. At the same time, the exponentially increasing number of known sequences is producing a corresponding increase in the demand for models. Structure models are becoming increasingly useful in many areas of experimental structural biology as well. Prediction of intrinsically disordered regions and domain boundaries, both areas assessed in CASP7,^14^,^16^ are critical to the design of constructs for protein overexpression. As noted earlier, it is now clear from the CASP7 results^12^ and other work^27^,^28^ that the best model structures should significantly increase the range of applicability of molecular replacement methods in crystallography. Structure modeling tools have been shown to play a critical enabling role in protein design.^29^ Talks at the CASP7 meeting also explored emerging application areas in cryoelectron microscopy, small angle X-ray scattering, and deducing structure from chemical crosslinks. Other potential application areas are in interpreting NMR data, and in refinement of crystal structures. The greater emphasis on methods for predicting the accuracy of a model^21^ in CASP7 also strongly reflects the increasingly practical and applied nature of the field, and inclusion of error estimates will make modeling a more respectable field, comparable with well evolved experimental areas. Indeed, methods for assessment of model accuracy in the structure-prediction field can be considered to be at a more advanced level than those currently employed in crystallography and NMR structure determination.

## THE IMPACT OF CASP

As discussed earlier, we have seen considerable progress in the accuracy of structure models during the course of the CASP experiments. It is hard to know how much of this would have occurred anyway, though naturally, as organizers, we would like to think that some of it is CASP driven. We do think it is true that it is now much clearer what methods work, and how well, and where the bottlenecks to progress are, and so where effort may most effectively be focused. One original motivation for CASP was that the peer reviewed publication system was not always performing as it should, and that, together with a lack of objective testing, resulted in a much higher rate of misleading claims making it to print than in most other disciplines. There has been a reduction in such claims, but they are far from being eliminated entirely.

A recognized downside of CASP is that it focuses attention on results, at the expense of methods. We have taken several steps to redress this imbalance. As noted above, including developing methods in the CASP meeting program was not successful. At the CASP6 meeting, we also introduced four “CASP Challenges”^6^ intended to focus attention on specific areas of methods development. Progress in systematically pursuing these has been slower than anticipated, but one “Off-CASP” experiment has already been held. This was “CASPR,” intended to allow predictors to explore the strengths and weaknesses of their methods for refining initial models towards the experimental structure. A set of best models from seven CASP5 and CASP6 targets were offered as starting structures, and predictors were invited to return refined versions, using the standard Prediction Center machinery of collecting predictions. These are of course not blind predictions—the experimental structures are all available. The results can be found on the Prediction Center web site. The experiment was generally considered informative and useful. We are planning to hold the second experiment, on modeling single residue mutations, shortly. Decoy sets will also shortly be released by the Prediction Center to encourage progress on the challenge of improving scoring functions for picking the most accurate models from a set of candidates, a current bottleneck, particularly in template-free modeling.

## FUTURE DEVELOPMENTS

There will be a CASP8 experiment, running from the Spring of 2008, and culminating in a meeting in December of that year. The meeting is planned to take place in Europe, as did CASP6. In general, future meetings will likely alternate between continents, reflecting the roughly equal and dominant participation of groups from each. We also plan to have a second “Between CASPs” meeting early in 2008, aimed at a broader audience. Also, as outlined above, the “Off-CASP” experiments will continue to be developed. Those interested in any of these areas should check the CASP web site for further announcements.
